# Nephroprotective mechanisms of Rhizoma Chuanxiong and Radix et Rhizoma Rhei against acute renal injury and renal fibrosis based on network pharmacology and experimental validation

**DOI:** 10.3389/fphar.2023.1154743

**Published:** 2023-05-09

**Authors:** Jun Li, Tonglu Li, Zongping Li, Zhiyong Song, Xuezhong Gong

**Affiliations:** Department of Nephrology, Shanghai Municipal Hospital of Traditional Chinese Medicine, Shanghai University of Traditional Chinese Medicine, Shanghai, China

**Keywords:** Chuanxiong–Dahuang herb pair, acute kidney injury, renal fibrosis, network pharmacology, experimental validation, p53 regulation

## Abstract

The molecular mechanisms of Rhizoma Chuanxiong (Chuanxiong, CX) and Rhei Radix et Rhizoma (Dahuang, DH) in treating acute kidney injury (AKI) and subsequent renal fibrosis (RF) were investigated in this study by applying network pharmacology and experimental validation. The results showed that aloe-emodin, (−)-catechin, beta-sitosterol, and folic acid were the core active ingredients, and *TP53*, *AKT1*, *CSF1R*, and *TGFBR1* were the core target genes. Enrichment analyses showed that the key signaling pathways were the MAPK and IL-17 signaling pathways. *In vivo* experiments confirmed that Chuanxiong and Dahuang pretreatments significantly inhibited the levels of SCr, BUN, UNAG, and UGGT in contrast media-induced acute kidney injury (CIAKI) rats (*p* < 0.001). The results of Western blotting showed that compared with the control group, the protein levels of p-p38/p38 MAPK, p53, and Bax in the contrast media-induced acute kidney injury group were significantly increased, and the levels of Bcl-2 were significantly reduced (*p* < 0.001). Chuanxiong and Dahuang interventions significantly reversed the expression levels of these proteins (*p* < 0.01). The localization and quantification of p-p53 expression in immunohistochemistry technology also support the aforementioned results. In conclusion, our data also suggest that Chuanxiong and Dahuang may inhibit tubular epithelial cell apoptosis and improve acute kidney injury and renal fibrosis by inhibiting p38 MAPK/p53 signaling.

## 1 Introduction

Acute kidney injury (AKI) is characterized by an abrupt loss of renal function, which mainly manifests as increased serum creatinine levels and decreased urine output (Khwaja, 2012). A meta-analysis showed that total morbidity and mortality rates of adult AKI were 21.6% and 23.9%, respectively (Susantitaphong et al., 2013). There remains no effective treatment for AKI, and prevention is currently the primary focus. While chemical and biological agents with beneficial effects on AKI have been reported, these are still in the preclinical research stage (Yang et al., 2016; Ronco et al., 2019). Regarding the pathological mechanism of AKI, due to pathological factors, various stress processes in the kidney affect renal tubular epithelial cells by causing oxidative stress damage, inflammation, necrosis, mitochondrial dysfunction, apoptosis, and autophagy (Sureshbabu et al., 2015; Cybulsky, 2017; Kimura et al., 2017). Although the causes of AKI include renal insufficiency, nephrotoxic drugs, and sepsis, their pathological mechanisms are related to hemodynamic changes, oxidative stress, and inflammation injury. In addition to an increased near-term risk of mortality, AKI patients also have a long-term risk of CKD (See et al., 2019). After the occurrence of AKI, if the kidney tissue is repaired excessively or incompletely or if the damage persists, renal dysfunction and renal fibrosis (RF) may occur (He et al., 2017). RF is a common pathway for all kidney injuries, leading to end-stage nephropathy, characterized by tubular atrophy, epithelial cell apoptosis, massive inflammatory cell infiltration, myofibroblast activation, and over-accumulation of the extracellular matrix (Dong et al., 2019; Livingston et al., 2022). Therefore, during the treatment of AKI and subsequent CKD, we have to consider the risk of the occurrence of RF and effective prevention methods. Rhizoma Chuanxiong (Chuanxiong, CX) is derived from the dried rhizome of *Conioselinum anthriscoides* “Chuanxiong” (Apiaceae). Rhei Radix et Rhizoma (Dahuang, DH) is mainly derived from the dried roots and rhizomes of *Rheum officinale* Baill. (Polygonaceae) and *Rheum palmatum* L. (Polygonaceae). Studies have shown that the chemical components in CX mainly include volatile oils, phenolic acids, alkaloids, and polysaccharides, which have good pharmacological activities on cardiovascular and cerebrovascular diseases and the nervous system, liver, and kidneys (Chen et al., 2018). The main chemical components of DH include anthraquinones, flavonoids, and ellagitannins, which have good value in anti-myocardial ischemia and have anti-tumor, anti-inflammatory, and antioxidant effects. In addition, the bound anthraquinone components in DH have laxative effects (Zhang et al., 2021). In terms of traditional Chinese medicine (TCM) theory, *C. anthriscoides* “Chuanxiong” (Apiaceae) (Chuanxiong, CX) and *R. officinale* Baill. (Polygonaceae) (Dahuang, DH) are frequently used to dissipate stasis, activate blood, and remove toxicity (Zheng et al., 2021; Zhou L. et al., 2022). A retrospective study from Taiwan, China, involving 14,718 patients with chronic kidney disease (CKD) showed that TCM, including DH, improved long-term survival in patients with CKD (Huang et al., 2018). CX and DH reportedly reduce the AKI caused by contrast media by inhibiting oxidative stress and regulating apoptosis (Gong et al., 2013). An herbal formula mainly composed of CX and DH also improved AKI in patients with CKD (Gong, 2018). In addition, our study confirmed that tetramethylpyrazine (a characteristic alkaloid of CX) improved AKI caused by arsenic toxicity and contrast media (Gong et al., 2016; Gong et al., 2019). In multiple clinical studies, a CX- and DH-based herbal formula was shown to affect the expression of a variety of RF-associated cytokines as well as improving AKI. This raised the question of whether CX and DH further intervene in the occurrence of RF after AKI. Thus, CX and DH are natural herbs with the potential to treat AKI and RF, and their mechanisms need to be investigated. In this study, we explored the molecular mechanisms of CX and DH intervention in AKI and RF by network pharmacology and experimentally verified in an *in vivo* model.

## 2 Materials and methods

### 2.1 Establishment of CX and DH active ingredients and targets

The relevant chemical components of CX and DH were retrieved from the Traditional Chinese Medicine Systems Pharmacology Database and Analysis Platform (TCMSP) and screened according to their pharmacokinetic characteristics (Ru et al., 2014). Target active ingredients with oral bioavailability (OB) ≥30% and drug-likeness (DL) ≥ 0.18 were identified (Dong et al., 2021). In addition, active ingredients that are used as quality control indicators for CX and DH medicinal materials in the Chinese Pharmacopoeia were also included. Simultaneously, the targets of related components were obtained from TCMSP, and the target proteins were converted into standard genes using the UniProt database (Consortium, 2021). For active ingredients without targets, we predicted the target genes of these small drug molecules through PharmMapper (Liu et al., 2010). Among the predicted target genes, the 10 genes with the highest fit scores were selected for subsequent analysis.

### 2.2 Establishment of AKI- and RF-related targets

Target genes of AKI and RF were obtained from the GeneCards database, DisGeNET database (Piñero et al., 2020), Online Mendelian Inheritance in Man (OMIM) database (Amberger et al., 2015), and Therapeutic Target Database (TTD) (Zhou Y. et al., 2022). The keywords used in the search included “acute kidney injury” and “renal fibrosis.” The targets identified in the four databases were combined to remove duplicates and standardize the final target names. The targets of CX and DH were crossed with the disease targets to obtain the core target of drug intervention in disease.

### 2.3 Establishment of a protein–protein interaction network

The protein–protein interaction (PPI) network comprises individual proteins that interact to participate in all aspects of life processes, including biological signal transmission, gene expression regulation, energy and material metabolism, and cell cycle regulation (Snider et al., 2015). After importing potential key target genes into the Search Tool for the Retrieval of Interacting Genes/Proteins (STRING) database (Szklarczyk et al., 2021), we constructed a PPI network to analyze the relationship between key target proteins. Each node represented a protein in the PPI network, and each edge represented a potential functional association between two target genes.

### 2.4 Construction of an “herb–component–target” network

Cytoscape version 3.7.1 was used to construct an “herb–component–disease–target” network to reflect the complex relationship between CX and DH, and the potential target genes of AKI and RF (Shannon et al., 2003). In the visual network, each node represented a compound and target genes, with the lines representing the intermolecular interactions between the compound and target genes. We analyzed the network topology parameters to identify the key compounds and target genes.

### 2.5 GO biological function annotation and KEGG pathway analyses

The potential targets were imported into the Database for Annotation, Visualization, and Integrated Discovery (DAVID) for GO biological process and KEGG pathway enrichment analyses (Huang da et al., 2009a). A threshold of *p*-value <0.05 was used to screen the top biological processes or signaling pathways and visualize the results in the R language.

### 2.6 Screening of core target genes and annotation of GEO expression profiles

Based on the “herb–component–target” network, PPI network, and KEGG analysis, key active ingredients and core target genes were selected. To further confirm the more specific core genes, we verified the expression of the genes in the Gene Expression Omnibus (GEO) database. First, we downloaded the gene set associated with AKI from the GEO database. After sample quality assessment, data standardization, batch effect removal, probe annotation, etc., differentially expressed gene analysis was performed using the limma package in R software (Ritchie et al., 2015). Finally, volcano plot and heatmap were drawn using the ggplot2 and pheatmap packages (Ito and Murphy, 2013).

### 2.7 Molecular docking verification of active ingredients and core target proteins

Based on the “herb–component–target” network, PPI network, and KEGG analysis, key active ingredients and core target proteins were selected. The structural files of the active ingredients and target proteins were downloaded from the PubChem and Protein Data Bank (PDB) databases, respectively (Goodsell et al., 2020; Kim et al., 2021). Discovery Studio v16 was used to process the active ingredient and target protein before docking and perform molecular docking. Finally, the binding activity of the active ingredient to the target protein was evaluated based on the docking score and energy.

### 2.8 Reagents and experimental animals

All chemicals were purchased from Sigma-Aldrich (St. Louis, MO, United States) unless otherwise stated. The contrast media iohexol (Omnipaque) was purchased from Amersham Health (Princeton, NJ, United States). A total of 32 adult 8–0-week-old male Sprague–Dawley rats weighing 200–250 g were purchased from the Shanghai Laboratory Animal Research Center. Rats were housed in an air-conditioned room at 23°C with a 12 h/12 h light/dark cycle. CX and DH were purchased from Shanghai Municipal Hospital of Traditional Chinese Medicine, Shanghai University of Traditional Chinese Medicine. The CX and DH herbal pair (CX 9 g and DH 18 g) was placed in a round-bottomed flask, and eight times the volume of pure water was added and steeped at room temperature for 60 min and boiled for 30 min. Then, the filtrate was filtered through gauze, and six times the volume of pure water was added to the residue and boiled for 30 min and filtered. The two aforementioned filtrates were then combined and placed in a rotary evaporator. After they were rotated, the filtrate was concentrated at 60°C until it contained 2.25 g of raw drug per mL and then set aside. Food and water were provided *ad libitum*, except for the day of dehydration. The animal study was reviewed and approved by the Medicine Animal Ethics Committee of Shanghai University of Traditional Chinese Medicine (Approval No. 2020025).

### 2.9 Induction of CIAKI and drug administration

A well-established rat model of CIAKI was used (Gong et al., 2013). A total of 32 rats were randomly divided into four groups of eight in each group: controls (CON), rats injected with CM (CIAKI), rats treated with CX and DH herbal pair (CXDH) and injected with CM (CIAKI + CXDH), and rats treated with 150 mg/kg/d N-acetylcysteine (NAC) and injected with CM(CIAKI + NAC). The CIAKI + CXDH group was filled with CXDH decoction every day for 7 days before molding, and the gavage amount was converted according to 50 times the normal amount of adult standard body weight (60 kg). The CIAKI + NAC group was given a daily dose of NAC (150 μg/g) intraperitoneally 3 days before molding. The CON and CIAKI groups were given daily gastric lavage with an equal volume of phosphate buffer 7 days before molding. The specific preparation method of the CIAKI model is as follows: SD rats were injected with a nitric oxide synthase inhibitor (NG-nitro-L-arginine methyl ester, L-NAME, 10 mg/kg, i.p.), followed after 15 and 30 min, respectively, by injection of an inhibitor of prostaglandin synthesis (indomethacin, 10 mg/kg, i.p.) and iohexol (1.5–2 g iodine/kg, i.p.). The CON group received injections of an equivalent volume of saline. Animals were euthanized 24 h after modeling, serum was obtained by blood collection from the tail vein, and kidneys were collected for biochemical and morphological examination. The study of urinary N-acetyl-β-glucosaminidase (UNAG) and urinary γ-glutamyl transpeptidase (UGGT) in 24-h urine samples was conducted on the same day.

### 2.10 Western blot analysis

Western blotting was performed as previously described (Gong et al., 2013). The primary antibodies used were as follows: anti-p38 MAPK (Cell Signaling Technology, 1:1000); anti-phospho-p38 MAPK (Cell Signaling Technology, 1:1000); anti-GAPDH (Cell Signaling Technology, 1: 1000); anti-Bcl-2 (Affinity Biosciences, 1: 1000); anti-Bax (Cell Signaling Technology, 1: 1000); anti-β-actin (Cell Signaling Technology, 1: 1000); anti-p53 (Cell Signaling Technology, 1: 1000); anti-phosphor-p53 (Affinity Biosciences, 1: 1000); HRP-labeled goat anti-rabbit IgG (Beyotime Biotechnology, 1: 1000), and HRP-labeled goat anti-mouse IgG (Beyotime Biotechnology, 1: 1000). All experiments were performed at least three times (i.e., three separate protein preparations) under the same conditions.

### 2.11 Statistical analysis

Results are expressed as mean ± SD. One-way analysis of variance (ANOVA) with Tukey’s *post hoc* multiple-comparison test was used to determine the significance of differences in multiple comparisons. Differences were considered significant if *p* < 0 05, highly significant if *p* < 0 01, and very highly significant if *p* < 0.001.

## 3 Results

### 3.1 Active ingredients and targets of CX and DH

Among 281 chemical components of CX and DH retrieved from the TCMSP database and screened based on pharmacokinetic characteristics (OB ≥ 30% and DL ≥ 0.18), 23 chemical components were included in the analyses. After including five characteristic compounds (emodin, chrysophanol, physcion, ferulic acid, and tetramethylpyrazine), we ended up with 28 active ingredients. In addition to OB and DL, the molecular name, molecular ID, molecular weight (MW), and half-life (HL) are shown in Table 1. Through combination and deduplication, we obtained 198 active ingredient targets of the 28 active ingredients. Information on the active ingredients and targets of CX and DH is shown in the Supplementary Material.

**TABLE 1:** Active ingredients of CX and DH.

Herb	Mol. ID	Molecule name	OB (%)	DL	MW	HL
Dahuang (Radix et Rhizoma Rhei)	MOL002293	Sennoside D_qt	61.05	0.61	524.50	33.92
	MOL002276	Sennoside E_qt	50.69	0.61	524.50	33.59
	MOL002303	Palmidin A	32.45	0.65	510.52	32.14
	MOL002268	Rhein	47.07	0.28	284.23	32.12
	MOL000471	Aloe-emodin	83.38	0.24	270.25	31.49
	MOL002288	Emodin-1-O-beta-D-glucopyranoside	44.81	0.79	432.41	29.79
	MOL002259	Physciondiglucoside	41.65	0.63	608.60	27.61
	MOL002280	Torachrysone-8-O-beta-D-(6′-oxayl)-glucoside	43.02	0.74	480.46	16.29
	MOL002251	Mutatochrome	48.64	0.61	552.96	15.73
	MOL002235	Eupatin	50.80	0.41	360.34	13.94
	MOL002297	Daucosterol_qt	35.89	0.70	386.73	6.12
	MOL002260	Procyanidin B-5,3′-O-gallate	31.99	0.32	730.67	5.98
	MOL000358	Beta-sitosterol	36.91	0.75	414.79	5.36
	MOL002281	Toralactone	46.46	0.24	272.27	3.55
	MOL000554	Gallic acid-3-O-(6′-O-galloyl)-glucoside	30.25	0.67	484.40	2.48
	MOL000096	(−)-Catechin	49.68	0.24	290.29	0.38
	MOL000472	Emodin	24.40	0.24	270.25	0
	MOL001729	Chrysophanol	18.64	0.21	254.25	0
	MOL000476	Physcion	22.29	0.27	284.28	0
Chuanxiong (Rhizoma Chuanxiong)	MOL000433	Folic acid	68.96	0.71	441.45	24.81
	MOL002140	Perlolyrine	65.95	0.27	264.30	12.62
	MOL002157	Wallichilide	42.31	0.71	412.57	6.848
	MOL001494	Mandenol	41.99	0.19	308.56	5.39
	MOL000359	Sitosterol	36.91	0.75	414.79	5.37
	MOL002135	Myricanone	40.59	0.51	356.45	4.39
	MOL002151	Senkyunone	47.66	0.24	326.52	2.42
	MOL000360	Ferulic acid	39.56	0.06	194.2	2.38
	MOL002202	Tetramethylpyrazine	20.01	0.03	136.22	0

### 3.2 AKI- and RF-associated target genes

A total of 7,453 AKI-related target genes and 6,581 RF-related target genes were retrieved in GeneCards, of which 1,258 and 668 target genes, respectively, met the screening criteria (relevance score ≥10). A total of 185 AKI-related target genes and 570 RF-related target genes were retrieved in DisGeNET, while nine AKI-related target genes and 14 RF-related target genes were retrieved in OMIM, and three AKI-related target genes and three RF-related target genes were retrieved in TTD. The target genes retrieved from the four databases were intersected and de-duplicated to obtain a total of 514 target genes. Specific target gene information for AKI and RF retrieved from the database is available in the Supplementary Material.

### 3.3 PPI network

**FIGURE 1:**
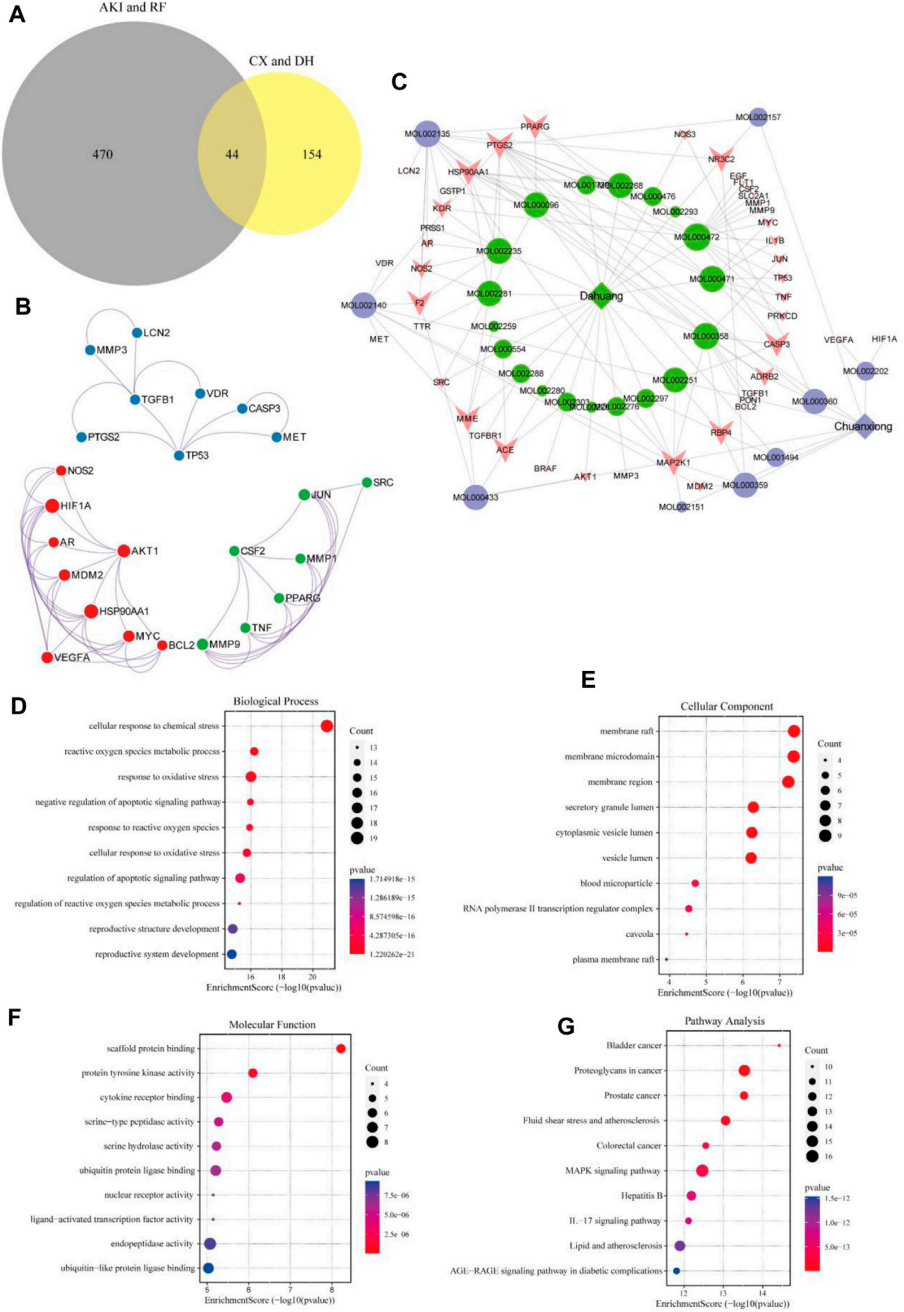
Target gene network construction and enrichment analysis. Venn diagram of drugs and disease genes **(A)**. PPI network of target genes **(B)**. “Herb–component–target” network **(C)**. GO and KEGG pathway enrichment analyses **(D–G)**.

A total of 44 potential targets were obtained after the intersection of disease and drug target genes (Figure 1A). The 44 target genes and their full names are shown in Table 2. These 44 target genes were sequentially imported into the STRING database to obtain a PPI network diagram (Figure 1B). Simultaneously, we used CytoHubba plugins to get the top 10 hub genes in terms of connectivity. The top 10 hub genes in the PPI network are tumor necrosis factor (*TNF*), vascular endothelial growth factor a (*VEGFA*), cellular tumor antigen p53 (*TP53*), transcription factor AP-1 (*JUN*), caspase-3 (*CASP3*), prostaglandin G/H synthase 2 (*PTGS2*), hypoxia-inducible factor 1 subunit alpha (*HIF1A*), epidermal growth factor (*EGF*), interleukin 1 beta (*IL1B*), and matrix metallopeptidase 9 (*MMP9*).

**TABLE 2:** Target genes in the network.

Gene	Name	Gene	Name
*TP53*	Tumor protein P53	*FLT1*	Fms-related receptor tyrosine kinase 1
*TNF*	Tumor necrosis factor	*MDM2*	MDM2 proto-oncogene
*ACE*	Angiotensin I-converting enzyme	*PTGS2*	Prostaglandin-endoperoxide synthase 2
*LCN2*	Lipocalin 2	*PPARG*	Peroxisome proliferator-activated receptor gamma
*IL1B*	Interleukin 1 beta	*MET*	MET proto-oncogene, receptor tyrosine kinase
*F2*	Coagulation factor II	*VDR*	Vitamin D receptor
*MYC*	MYC proto-oncogene, bHLH transcription factor	*SRC*	SRC proto-oncogene, non-receptor tyrosine kinase
*VEGFA*	Vascular endothelial growth factor A	*GSTP1*	Glutathione S-transferase pi 1
*TGFB1*	Transforming growth factor beta 1	*KDR*	Kinase insert domain receptor
*CASP3*	Caspase 3	*RBP4*	Retinol-binding protein 4
*NOS3*	Nitric oxide synthase 3	*PRKCD*	Protein kinase C delta
*MMP9*	Matrix metallopeptidase 9	*ADRB2*	Adrenoceptor beta 2
*AKT1*	AKT serine/threonine kinase 1	*NR3C2*	Nuclear receptor subfamily 3 group C member 2
*TTR*	Transthyretin	*MMP1*	Matrix metallopeptidase 1
*EGF*	Epidermal growth factor	*PON1*	Paraoxonase 1
*BRAF*	B-Raf proto-oncogene, serine/threonine kinase	*MMP3*	Matrix metallopeptidase 3
*NOS2*	Nitric oxide synthase 2	*HSP90AA1*	Heat shock protein 90 alpha family class A member 1
*MME*	Membrane metalloendopeptidase	*MAP2K1*	Mitogen-activated protein kinase 1
*BCL2*	BCL2 apoptosis regulator	*SLC2A1*	Solute carrier family 2 member 1
*CSF2*	Colony-stimulating factor 2	*PRSS1*	Serine protease 1
*HIF1A*	Hypoxia-inducible factor 1 subunit alpha	*TGFBR1*	Transforming growth factor beta receptor 1
*JUN*	Jun proto-oncogene, AP-1 transcription factor subunit	*AR*	Androgen receptor

### 3.4 “Herb–component–target” network

To deeply explore the relationship between the active ingredients, core targets, and disease targets of CX and DH, we used Cytoscape to build an “herb–component–target” network (Figure 1C). The network contained 72 nodes, of which 28 represented active ingredients, 44 represented target genes, and 140 represented internode interaction relationships. The larger the area of the node, the more critical the position of the target or compound represented by this node in the network. The degree value was set as the screening condition. The active ingredients with a high core degree in CX are MOL002135, MOL000359, MOL002140, and MOL000433. The active ingredients with a high core degree in DH are MOL000472, MOL000358, MOL002235, MOL000471, and MOL000096. Genes with high connectivity in the network are *PTGS2*, *MAP2K1*, *HSP90AA1*, *RBP4*, *PPARG*, *MME*, *CASP3*, *NR3C2*, etc.

### 3.5 GO biological function annotation and KEGG pathway analyses

To further explore the mechanism of CX and DH in the treatment of AKI and RF, we performed GO biological function annotation and KEGG signaling pathway analyses of the 44 target genes (Huang da et al., 2009b). GO enrichment analysis was divided into biological process (BP), cellular component (CC), and molecular function (MF). The top 10 significantly enriched gene biological function catalogs for each part were used to generate histograms (Figures 1D–F). The BPs involved in these genes mainly included regulation of the apoptotic signaling pathway, negative regulation of the apoptotic signaling pathway, and response to oxidative stress. The CCs mainly included membrane raft, membrane microdomain, and secretory granule lumen. The MFs mainly included scaffold protein binding, protein tyrosine kinase activity, and cytokine receptor binding. In total, 135 signaling pathways were obtained through KEGG pathway enrichment analysis, of which the first 10 are shown in bubble diagrams (Figure 1G). As shown in Figure 1G, multiple signaling pathways were involved in the CX and DH intervention in AKI and RF, including the mitogen-activated protein kinase (MAPK) and interleukin 17 (IL-17) signaling pathways. Compared with the adjusted *p*-value and the number of enriched genes, the MAPK signaling pathway is undoubtedly the most important of these pathways.

### 3.6 Target protein screening and molecular docking

**FIGURE 2:**
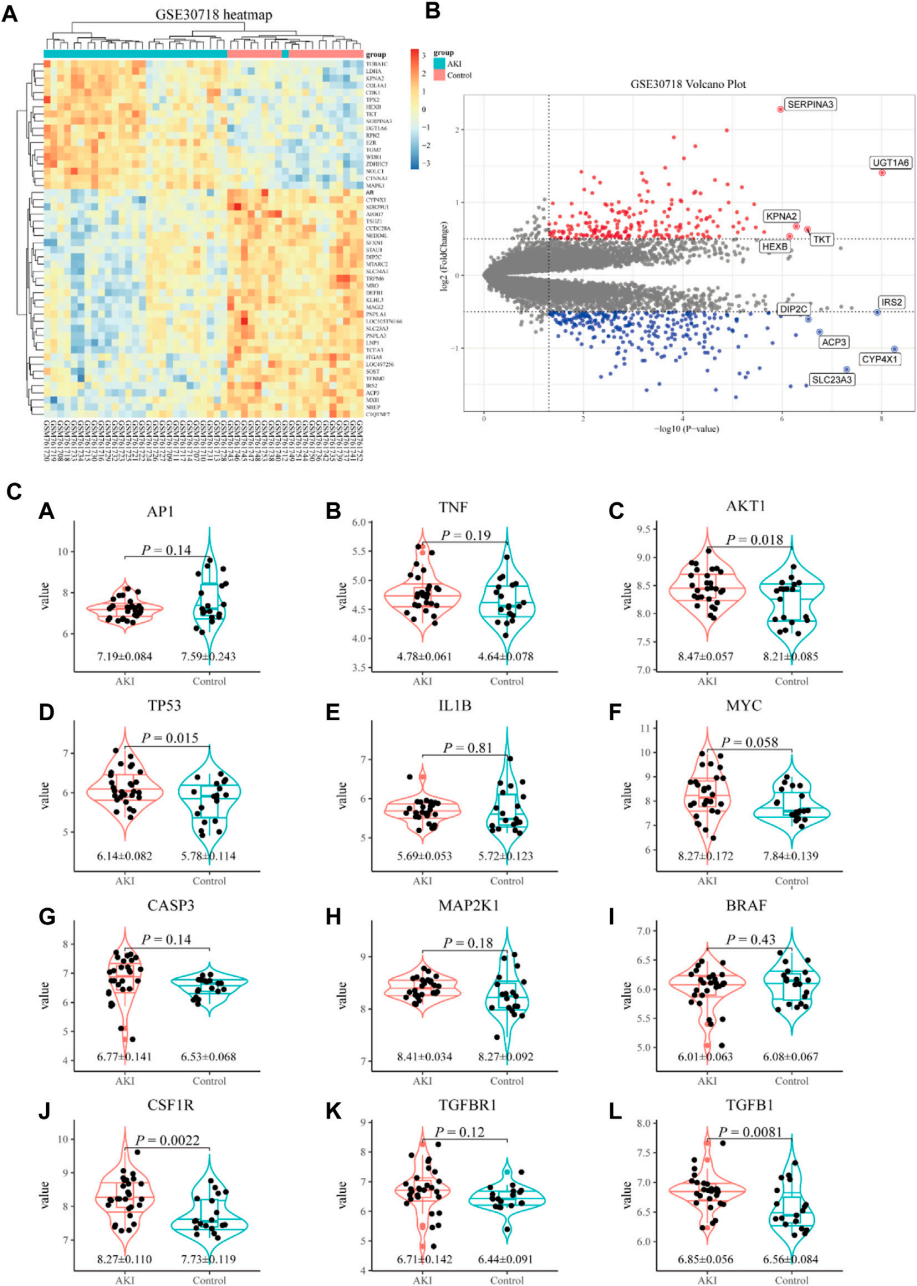
Screening of key target genes based on the AKI gene expression dataset GSE30718. Heatmap and volcano plot of GSE30718 **(A,B)**. Expression of the hub gene in the MAPK signaling pathway **(C)**. Compared with the control group, the expression of *AP1* **(A)**, *TNF* **(B)**, *AKT1* **(C)**, *TP53* **(D)**, *IL1B* **(E)**, *MYC* **(F)**, *CASP3* **(G)**, *MAP2K1* **(H)**, *BRAF* **(I)**, *CSF1R* **(J)**, *TGFBR1* **(K)**, and *TGFB1* **(L)** in the AKI group.

**FIGURE 3:**
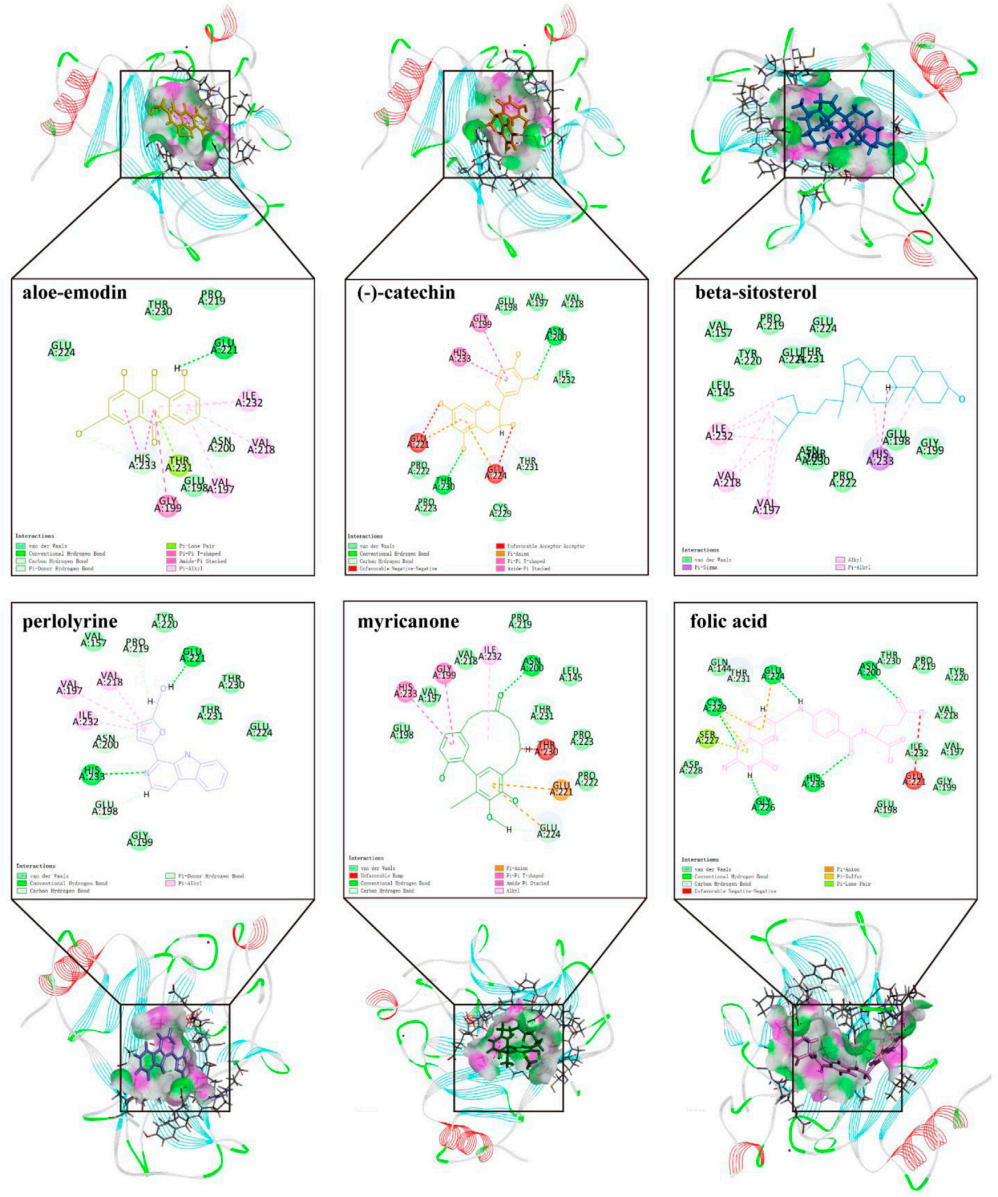
Results and details of molecular docking.

To further screen for more specific target genes, we downloaded the AKI-related GSE30718 dataset from the GEO database that contains expression profiling data from 28 AKI patients and 11 control patients (Famulski et al., 2012). Concurrently, we visualized the differential expression of genes in the GSE30718 gene set by heatmap and volcano plot (Figures 2A, B). Finally, the expression of key target genes in the MAPK signaling pathway was visualized in the dataset (Figure 2C). Figure 2C shows that the expression of the *TP53*, *AKT1*, *CSF1R*, and *TGFBR1* genes in the AKI group was significantly different from that in the control group (*p* < 0.05). Since *TP53* is one of the most connected genes in the top 10 hub genes, we selected p53 encoded by *TP53* as the target protein for subsequent molecular docking. The PDB ID of p53 is 6SL6 (Langenberg et al., 2020). We also chose the nine most important active ingredients in the “herb–component–target” network as small-molecule objects for docking. The structures of these small drug molecules were obtained—aloe-emodin, (−)-catechin, beta-sitosterol, eupatin, toralactone, perlolyrine, myricanone, and folic acid (three from CX and five from DH). The receptor target protein and small drug molecules were processed using Discovery Studio v16 software for docking, and a three-dimensional map of the docking results is shown in Figure 3. The docking score and absolute energy of the docking results are shown in Table 3. The results showed that folic acid, beta-sitosterol, and (−)-catechin had better docking effects and may have a potential intervention effect on p53.

**TABLE 3:** Docking results of molecules and p53.

Herb	Mol. ID	Molecule name	Absolute energy	Relative energy	LibDockScore
DH	MOL000472	Emodin	0	0	0
DH	MOL000358	Beta-sitosterol	47.7335	12.0619	91.4289
DH	MOL000471	Aloe-emodin	37.18	0	74.4522
DH	MOL002235	Eupatin	0	0	0
DH	MOL002281	Toralactone	0	0	0
DH	MOL000096	(−)-Catechin	32.0703	3.2717	88.5165
CX	MOL002135	Myricanone	86.067	10.2008	65.5274
CX	MOL002140	Perlolyrine	142.725	0.00876478	84.1469
CX	MOL000433	Folic acid	47.6251	4.42251	104.357

### 3.7 CX and DH improved kidney injury in CIAKI rat models

**FIGURE 4:**
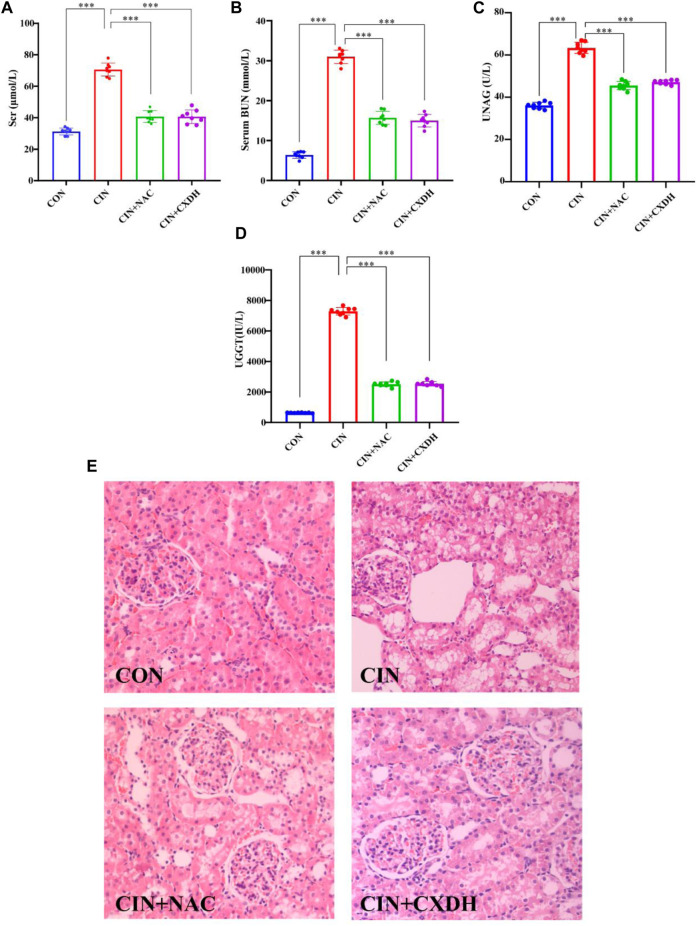
CX and DH protected the kidney from damage in CIAKI rats. The serum levels of creatinine **(A)**, blood urea nitrogen **(B)**, urinary N-acetyl-β-glucosaminidase **(C)**, and urinary γ-glutamyl transpeptidase **(D)** were examined using automated biochemistry assays. Photomicrographs (original magnification, ×400) illustrate hematoxylin and eosin staining of the kidney tissues from rats in the following groups **(E)**: CON, CIAKI, CIAKI + NAC, and CIAKI + CXDH. Figures are representative of 5–8 rats from each group. Data are represented as means ± standard deviation (SD; *n* = 5). *p < 0.05, **p < 0.01, ***p < 0.001.

As shown in Figures 4A–D, after 24 h of molding, compared with the CON group, the SCr, BUN, and UNAG levels were markedly elevated (*p* < 0.001). Both CXDH and NAC pretreatments significantly inhibited SCr, BUN, UNAG, and UGGT in CIAKI rats, and the effects are similar (*p* < 0.001). Histological analysis (Figure 4E) showed that compared with the CON group, the CIAKI group had severe tubulointerstitial damage, including significant tubular epithelial cell swelling and pronounced vacuolar change, and the glomeruli were basically normal. After treatment with CXDH and NAC, these signs of tissue damage were significantly alleviated.

### 3.8 CX and DH inhibit the p38 MAPK/p53-mediated apoptosis cascade in CIAKI rats

**FIGURE 5:**
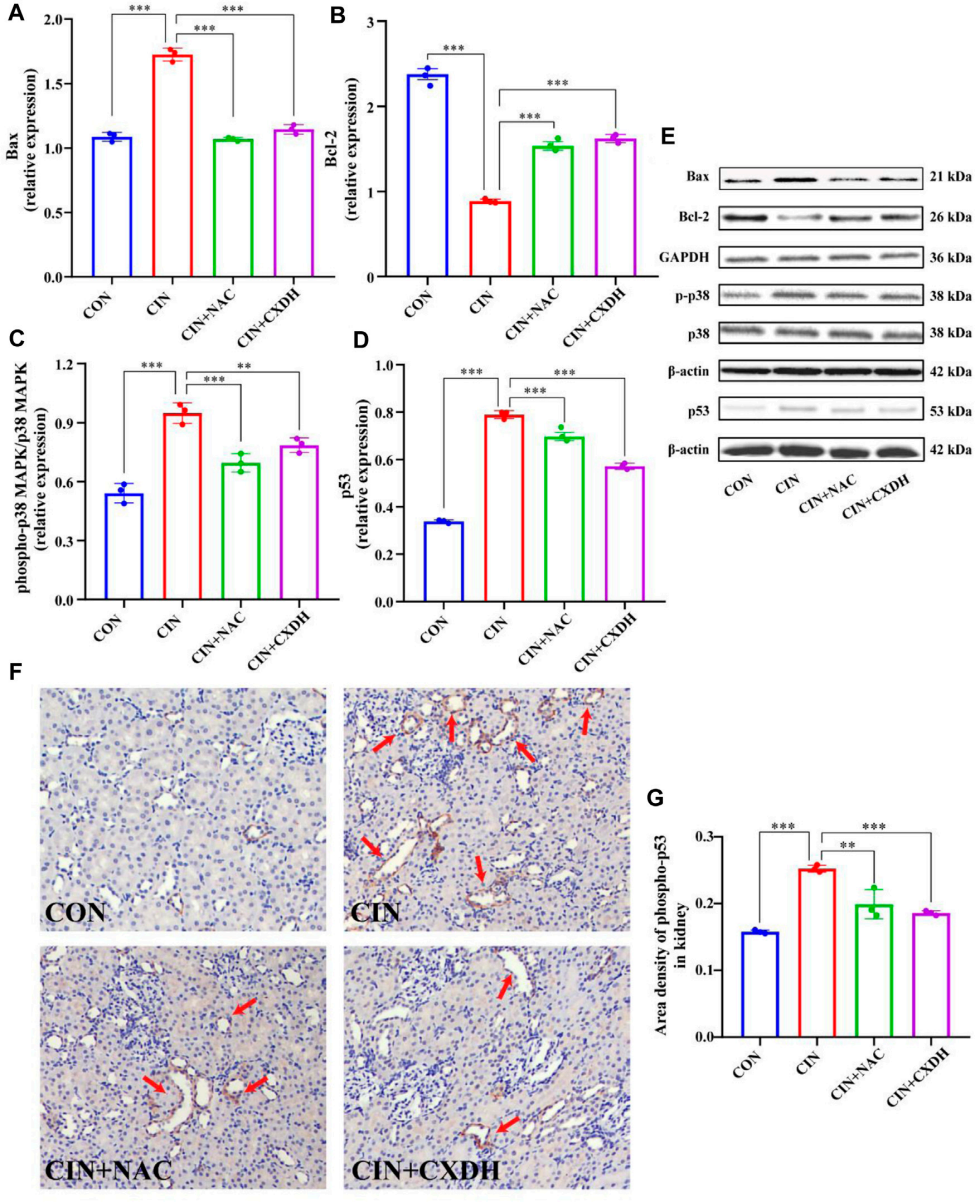
CX and DH inhibit the p38 MAPK/p53-mediated apoptosis cascade in CIAKI rats. The abundance of Bcl-2 **(A)** and Bax **(B)** was quantified by densitometry and normalized to that of GAPDH. Phospho-p38 MAPK and total-p38 MAPK expression levels by Western blotting (*n* = 3 each) **(C)**. Total-p53 expression by Western blotting (*n* = 3 each) **(D)**. Bcl-2, Bax, phospho-p38 MAPK, total-p38 MAPK, and p53 expression levels by Western blotting (*n* = 3 each) **(E)**. IHC staining of phospho-p53 in CON, CIAKI, CIAKI + NAC, and CIAKI + CXDH groups, respectively **(F)**. Note the positive-stained area (yellow; color refers to the online version only) of IHC staining (arrow). Semiquantitative analysis of phospho-p53 expression in kidneys with IHC **(G)**. Data are shown as means ± SD (*n* = 3 each). *p < 0.05, **p < 0.01, ***p < 0.001.

To further verify the effect of CXDH on p38 MAPK/p53 signaling, the expression levels of p38 MAPK, phospho-p38 MAPK (p-p38), p53, Bax, and Bcl-2 were determined by enzyme-linked immunosorbent assay. As shown in Figures 5A–E, the protein levels of p-p38/p38 MAPK, p53, and Bax in the CIAKI group were significantly increased, and the levels of Bcl-2 were significantly decreased (*p* < 0.001). After CXDH intervention, the expression levels of all these proteins were reversed. The results of immunohistochemistry (IHC) techniques in Figure 5F showed that the positive expression range of p-p53 in the kidney tissue of CIAKI rats was large and the color was brown compared to the CON group. Conversely, after CXDH intervention, the staining range and depth of positive expression of p-p53 protein were reduced. The results of IOD/area analysis (Figure 5G) showed that the expression of p-p53 protein in the CIAKI group was significantly increased compared to that in the CON group, and this trend was significantly reversed after CXDH administration (*p* < 0.001). Taken together, all these findings suggest that CXDH inhibits the expression of p38 MAPK/p53-mediated apoptosis cascade in CIAKI rats and attenuates acute injury to renal tissue.

## 4 Discussion

AKI is an acute kidney disease characterized by acute changes in renal function (Ferenbach and Bonventre, 2015). So far, the incidence and disease burden of AKI are still increasing (Xu et al., 2015; Sawhney and Fraser, 2017). Therefore, there is an urgent need to develop effective treatment methods for AKI. AKI pathogenesis is closely related to oxidative stress, inflammatory injury, apoptosis, and activation of autophagy pathways (Kimura et al., 2011; Linkermann et al., 2013; Rabb et al., 2016; Kusirisin et al., 2020). The results of the present study revealed that the 28 active ingredients of CX and DH play an important role in AKI treatment and are related to a variety of proteins and signaling pathways, suggesting their potential research value. The “herb–component–target” network showed that many target genes in AKI and RF are regulated by a variety of compounds. These genes included, but were not limited to, *AKT1*, *BCL2*, *CASP3*, *IL1B*, *JUN*, *MAP2K1*, *MDM2*, *MMP3*, *MYC*, *PPARG*, *PTGS2*, *TGFBR1*, *TNF*, and *TP53*. Enrichment analysis showed that the regulation of apoptotic signaling pathway, epithelial cell migration, response to oxidative stress, and MAPK and IL-17 signaling pathways are key parts of the mechanism.

**FIGURE 6:**
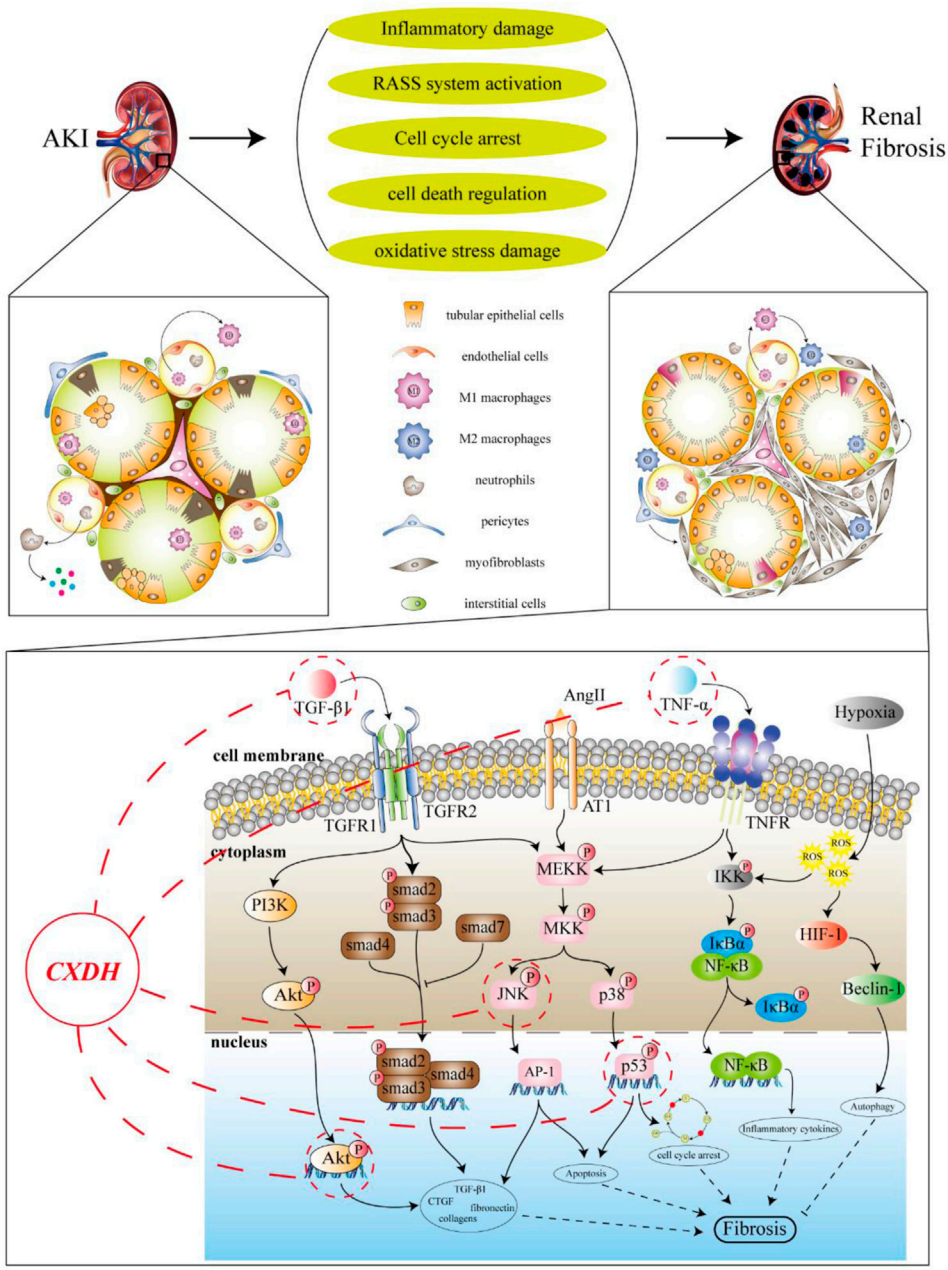
Target prediction of CX and DH intervention in RF. The progression of AKI to CKD is a complex process involving a variety of pathological processes including inflammatory injury, RASS system activation, cell cycle arrest, cell death, and oxidative stress injury, which can eventually lead to or worsen RF. Behind the fibrosis process, a variety of pathways and signaling molecules are activated, such as TGF-β/smad, MAPK, NF-κB, and PI3K/Akt signaling pathways. According to the target prediction results, it is speculated that CX and DH may reduce RF after AKI by regulating molecules such as p53 and Akt.

The progression of AKI to CKD is a complex process involving the regulation of multiple cellular and signaling pathways, including inflammatory injury, cell cycle arrest, and cell death regulation, which can ultimately lead to or aggravate RF (Figure 6) (He et al., 2017; Sato et al., 2020). During the acute phase of AKI, the secretion of cytokines and chemokines by the renal tubular epithelial cells (RTECs) increases, leading to interstitial inflammatory cell infiltration. Furthermore, damaged proximal tubules can stimulate the proliferation of macrophages and alter the infiltration of inflammatory cells in the renal interstitium, including driving the transformation of M1 to M2 macrophages (Meng et al., 2014). Chronic hypoxia and inflammatory responses are closely related, and long-term sustained activation of HIF may play a key role in initiating and promoting RF by regulating multiple signaling pathways in CKD (Ullah and Basile, 2019). After renal tubular injury, cell cycle arrest in a certain phase can disrupt the normal injury repair processes. The proportion of RTECs in G2/M arrest is closely related to the degree of fibrosis (Canaud and Bonventre, 2015). The internal regulation of cell death is also involved in the development of RF. When AKI injury persists, endothelial cell apoptosis reduces the peritubular microvessel density of the renal interstitium, resulting in chronic ischemia, hypoxia, and a persistent inflammatory response in the renal interstitium, ultimately leading to RF. Autophagy also plays a bidirectional regulatory role in the conversion of AKI to CKD. Moderate autophagy may protect cells by removing damaged protein aggregates and organelles, whereas excessive autophagy could damage the kidney and promote the occurrence of RF (Shi et al., 2016; Wang et al., 2020). Additionally, the activation of the renin–angiotensin system and mitochondrial damage are both involved in RF.

According to the aforementioned results, CX and DH may improve AKI and subsequent RF by regulating downstream apoptosis through the p38 MAPK/p53 pathway. The MAPK signaling pathway can transduce related extracellular stimulus signals into the cell and nucleus through the three-level kinase cascade pathway and participate in cell proliferation, growth, apoptosis, and other processes (Zhang and Liu, 2002). This pathway is highly evolutionarily conserved and can be divided into four subfamilies: ERK, p38 MAPK, c-Jun amino-terminal kinase (JNK), and ERK5, to form a parallel MAPK signaling pathway. Among these, p38 MAPKs and JNK are involved in the treatment of AKI (Kanellis et al., 2010; Cuarental et al., 2019). p38 MAPKs and JNK have similar functions, participate in various inflammation and stress signal transduction pathways, and regulate cell apoptosis and growth. CIAKI has become a major cause of hospital-acquired AKI (Keaney et al., 2013). Studies conducted by our team and other laboratories have confirmed that apoptosis induced by contrast agents through the p38 MAPK pathway is an important pathogenic mechanism in CIAKI (Gong et al., 2010; Quintavalle et al., 2011). Therefore, p38 MAPK is a promising therapeutic target for CIAKI, and we sought to explore its associated upstream and downstream reaction elements. Apoptosis is one of the downstream effects of p38 MAPK, which is a biochemical cellular breakdown process mediated by a specific set of proteins that interact with and programmed death-induced signals (Kønig et al., 2019). When a cell receives an apoptosis signal, it activates the initial cascade through different signaling pathways and degrades related substrates, leading to apoptosis. Due to the presence of multiple apoptosis signaling pathways, the Bcl-2 family mainly regulates cell apoptosis through the mitochondrial pathway (Chota et al., 2021). Intrarenal stress and ischemia both increase the Bax/Bcl2 ratio, which is the main determinant of cell death (Liu and Baliga, 2005). p53 is one of the elements of the downstream reaction of p38 MAPK and JNK and is also a target of interest in this study. p53 represents a family of tumor suppressors and is a key component of the cell’s response to stress (Kruiswijk et al., 2015; Ong and Ramasamy, 2018). Recent experimental studies have provided evidence to support the involvement of p53 in the development of AKI and subsequent renal repair primarily by modulating apoptosis, cell cycle arrest, and so on. Inhibition of the p53-signal-mediated apoptosis process may be an effective strategy to improve tubular injury in AKI (Liu et al., 2016; Ding et al., 2021). Consistent with the findings of previous studies, we observed an increase in p38 MAPK and p53 in the kidney tissue of rats in the model group. The results showed that CX and DH reduced the expression levels of p-p38/p38 MAPK and p53 in rats with CIAKI and regulated Bcl-2 and Bax to inhibit RTEC apoptosis. In addition to apoptosis, p53 activation leads to cell cycle block that may also promote RF after AKI. The expression of p21 is upregulated after p53 activation, and high levels of p21 lead to the downregulation of a large number of cell cycle genes, which, in turn, leads to cell cycle arrest (Engeland, 2022). Studies have shown that hypoxia-induced upregulation of p53 inhibits the expression of cyclin-dependent kinase 1 (CDK1), cyclin B1 (CyclinB1), and cyclin D1 (CyclinD1), resulting in accumulation of cells at the G2/M phase and activation of profibrotic TGF-β-mediated signaling pathways (Liu et al., 2019). In the ischemic, toxic, and obstructive models of AKI, p53 inhibitors can alleviate G2/M arrest and delay the development of RF (Yang et al., 2010). Nonetheless, the role of p53 in contrast or sepsis-induced AKI, two common forms of AKI in hospitalized patients, remains poorly understood (Tang et al., 2019). Therefore, exploring the role of p53 is of practical significance in CIAKI. In summary, CX and DH may inhibit RTEC apoptosis and improve AKI and subsequent RF by inhibiting p38 MAPK/p53 signaling. Meanwhile, based on the “herb–component–target” network, MOL000471 (aloe-emodin) and MOL000472 (emodin) can be found to have better targeting on p38 MAPK/p53 signaling. Therefore, they can provide a material basis for further mining of efficient herbal metabolites.

## 5 Conclusion

The MAPK signaling pathway plays an important role in acute kidney injury and subsequent RF. The present study emphasizes that inhibition of renal tubular epithelial cell apoptosis via p38 MAPK/p53 signaling may be an important renoprotective mechanism of CX and DH. Nevertheless, the present study is still incomplete, and the mechanisms of intervention for renal fibrosis require further evaluation in future studies.

## Data Availability

The datasets presented in this study can be found in online repositories. The names of the repository/repositories and accession number(s) can be found in the article/Supplementary Material.
