# Supplementary figures and images for: Nephroprotective mechanisms of Rhizoma Chuanxiong and Radix et Rhizoma Rhei against acute renal injury and renal fibrosis based on network pharmacology and experimental validation
Source: Front Pharmacol. 2023 May 9;14:1154743. doi: 10.3389/fphar.2023.1154743 (PMC10203597; doi:10.3389/fphar.2023.1154743)

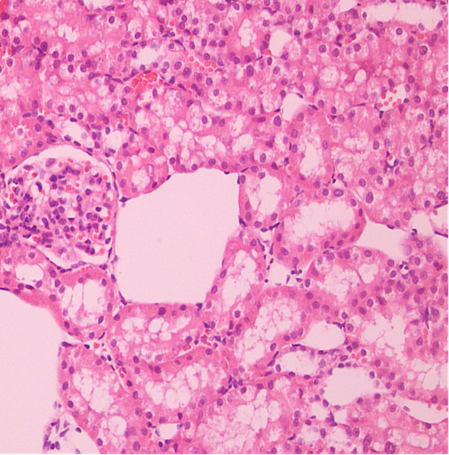

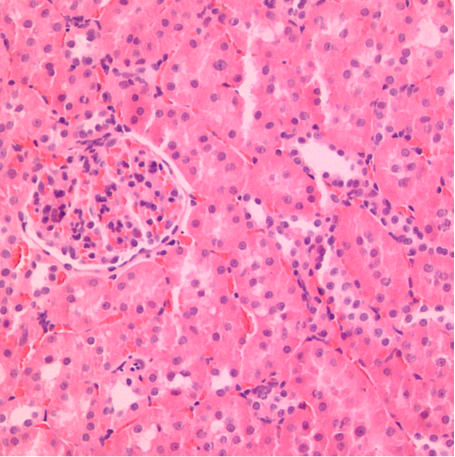
CON CIN


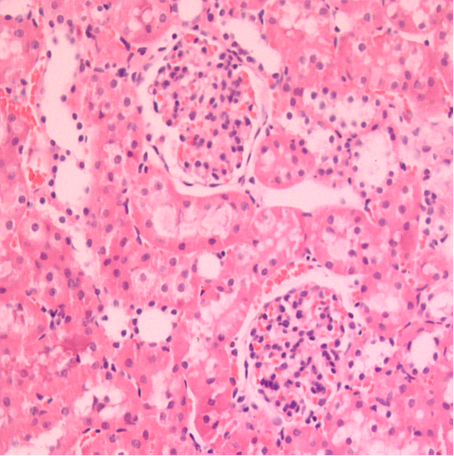

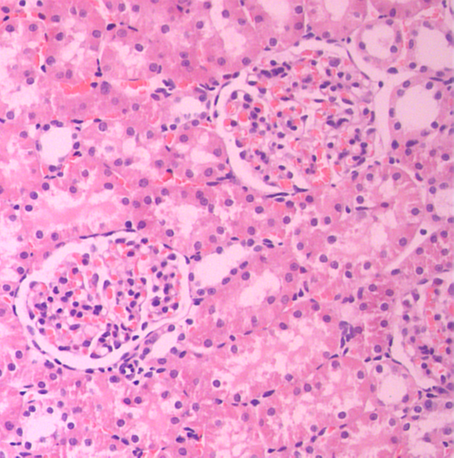
CIN+NAC CIN+CXDH

­

Supplement: Supplementary file 6 [file Table15.DOCX]
